# The Dragon and the Tiger: Realties in the Control of Tuberculosis

**DOI:** 10.1155/2012/625459

**Published:** 2012-07-04

**Authors:** P. Bhatter, A. Chatterjee, N. Mistry

**Affiliations:** Department of Tuberculosis, The Foundation for Medical Research, 84-A, R.G. Thadani Marg, Worli, Mumbai 400018, India

## Abstract

India and China are two Asian super-powers with developing economies carried on the shoulders of their booming populations. This growth can only be sustained by nurturing their “human resource”. However increasing reports of insufficient public health (PH) initiatives in India when compared to the aggressive PH system of China may prove to be the Achilles' heels for India. This review compares the PH system in India and China for combating Tuberculosis (TB), the disease responsible for maximum mortality and morbidity by a single infectious agent. While China has acknowledged the disease load and thereafter has methodically improved its reporting, detection, diagnosis and treatment, India is still in denial of the imminent health risk. The Indian PH system still considers TB as a “facultative” disease for which the required control measures are already in place and functioning. Globally, India and China recorded the highest Multi-Drug Resistant TB (MDR) cases notified in 2010 (64000 and 63000, respectively). Additionally non-government sources reported extremely high proportions of MDR in India. Here we have compared the medical, social and economic approaches of the two nations towards better management and control of TB. Does India have lessons to learn from China?

## 1. Introduction

The Tiger and the Dragon have been pitted against each other for a while now, as both countries have asserted their right-full place on the world stage, economically and otherwise. India's economic growth, hovering around 8-9% per year, has fuelled speculation on whether and when India may catch or surpass China's over 10% growth rate [1]. While India made its presence felt by a booming “skilled-labour-middle-class” bringing the technology revolution to its door steps, China has muscled its way through an “organized-labour-lower-middle-class” propelling industry and production. The immediate byproduct of the two different growth stories is the neglected rural population and “the taken for granted” urban population.

The focus on the rural population to usher economic reforms (which was the turning point in China's economic development) has constantly attempted to achieve social objectives such as education and health care which have brought to China a holistic development. On the contrary, the “shining India” story has resulted in greater disparity between the rural and the urban [1].

The differences between India and China are, however, beyond the omnipresent economic growth. The strategic reforms (political and social) in China have placed it in a better position as compared to India. Factually, statistics reveal that life expectancy at birth in China is 73.5 years; in India it is 64.4 years. The infant mortality rate is 50 per thousand in India compared with just 17 in China. China's adult literacy rate is 94%, compared with India's 74%. Only 66% of Indian children were immunized with triple vaccine (diphtheria/pertussis/tetanus), as opposed to 97% in China. Government expenditure on health care in China is nearly 5 times that in India [1].

The burning issue which is often downplayed is the emerging burden of MDR/XDR TB in the 2 nations. It has been more than 2 decades of running National Tuberculosis Programs (NTPs) for control of tuberculosis in both the countries. Yet, India and China rank first and second in the global burden of MDR TB [2]. While China's NTP has been termed as a model NTP for nationwide prevalence surveys, a sample vital registration system and a web-based case notification system [3], India is yet to implement any of these systems.

This review attempts to compare differences between the 2 nations in combating the single largest killer infectious disease in their territories. Simple steps like notification and surveillance, to complex issues of public-private mix, spurious drugs, and malnourishment have been addressed and the efforts on the part of the two countries towards the same have been elaborated.

## 2. Acknowledgment of the Problem versus the Politics of Denial

Of the 37 notifiable communicable diseases in China, TB ranks first in terms of notified cases and deaths [5]. The central government began efforts to revitalize its TB control program in 2000 with a strong political commitment to tackle TB. The concept of acceptance of the problem, identifying its requirement and the political will of TB eradication, has set China on a progressive path.

The Ministry of Health (MOH) in the Government of India (GOI) on the other hand does not have a centralized list of notifiable diseases. Each state in the country has its own list and the priorities change with the state. For Maharashtra [6] and Tamilnadu [7], TB is 12th and 11th on its priority list of 24 notifiable diseases. The culture of denial is so deep rooted that, in its first ever report on public health to the people submitted in the year 2010 [8], TB has been reported to have shown decreasing trends while several nongovernmental sources have reported MDRTB in excess of 25% [9, 10]. The under-reporting of the problem by the health system is further demonstrated by recent report of totally drug resistant (TDR) TB by a tertiary health care center in Mumbai [11]. The issue is not only adequate measures to eradicate TB, but to identify and accept that TB is a substantial problem for our country.

With this perspective, we have to mention that Indian democracy is still immature in delivering an effective, functional political system. The mechanisms for converging and coordinating across ministries, state and central governments, and peripheral health governance structures are virtually absent or weak. This is unlike China (a comparatively dictatorial society), where rules have been enforced more stringently than in India. To China's benefit and India's dismay, at least in the health sector and specifically TB control, the nonnegotiable government stand in China has harvested some advantage.

## 3. The Health Delivery System: Chinese CDC Standing Tall against Indian IDSP

### 3.1. Centre for Disease Control and Prevention (CDC)

The CDC in China is based on the example of CDC, Atlanta, GA, USA. The first such centre in China was established in 1998 in Shanghai. This model program was the precursor to a Chinese CDC created in Beijing in January 2002 and in 28 other regional provinces [12].

The creation of these centers indicates a policy response to China's changing disease patterns, perception of disease, and the governmental changes [12]. CDC acts as a central public health organization with integrated responsibility for both community and individual health.

The CDC carries out 4 main functions:

- make recommendations for public health policy and planning,
- surveillance,
- research on preventive medicine and health care services,
- provision for training and health care services.

The performance of CDC is spoken of by the mortality percentages, which have reduced from 36.98% in 1952 to 2.31% in 2001 for the infectious diseases. Of the infectious diseases that once dominated China, 12 have been eliminated in Shanghai [12]. However, the surveillance system in place has allowed keeping a track of the rising case of syphilis, gonorrhea, and TB. This allows tracing the changing disease dynamics and thus bringing in effective policy changes, which is not possible without an active surveillance mechanism.

### 3.2. Integrated Disease Surveillance and Planning (IDSP)

IDSP is a decentralized, state-based program launched in India by the MOH in November 2004. The main objectives of the program are (1) integrating and decentralization of surveillance activities; (2) strengthening of public health laboratories; (3) human resource development—training of state surveillance officers, district surveillance officers, rapid response team, other medical and paramedical staff; and (4) use of information technology for collection, collation, compilation, analysis, and dissemination of data [13].

The project was to achieve a national coverage in 2007 through a state wise expansion in a 3-phased manner. However, reports indicate to the contrary, and the project was running a year late in 2008 [14]. The problem lay with the fact that health and health issues are a subject of the state (so as to ease the load and amend the rules as per the requirement), but in effect it just gives more diversity. There is no common agenda, and each state ends up having its own program. This leads to variable performances of the health program in different states that are least effective in containing infectious diseases which are not bound by political and geographical boundaries.

CDC managed to achieve what it had promised—disease handling, national coverage, active surveillance—all of this in changing demographics. The objectives of IDSP are not to be criticized but its lacunae or its failures will be brought to light only when the project runs to a full scale. Integration and delivery of medical services from a common source not only brings about equality of treatment but also helps address issues like, ineffective drugs, loss of patients to private sector, and unmanageable disease scenarios.

## 4. Finance

A successful programme needs to acquire, utilize, and manage incoming funds to assess impact of resources on the population health and health systems. An unhindered yet regulated flow of money ensures sufficient and justifiable services as demanded by the system.

The total budget for TB control in China is around USD 238 million, an approximate double of that of India. The government funds around 86% of this budget and the remaining 14% is drawn from global funds. This enables China to input a little over US $200 to treat per patient which is 4 times the cost that India spends per patient [2]. This high budgetary allocation may be an added burden to the running NTP but ensures patient adherence, treatment completion and sufficient compensation for the staff.

The total budget for TB control in India for the year 2010 is around USD 100 million which has doubled in the last 5 years India. Of this a major fraction is contributed by loans, global funds, and grants while the national contribution remains a meager 1% of the total. Along with low government contribution in expenditure for TB control, India also has the lowest budget allocated in comparison to the four high burden countries [2].

As per the 2010 statistics, India spends US $50 on treating a TB patient using DOTS and USD 4000 on an MDRTB patient. Thus with an annual budget of USD 112 million, the treatment of 1982628 TB patients (USD 99,131,400) and 131000 MDR TB patient (USD 524,000,000) would require USD 623,131,400 which is a shortfall of USD 511,131,400.

The paucity of fund allocation towards TB control brings to light the lack of seriousness of the issue to the government. It also raises questions whether the funds are sufficient to buy enough stocks of medicines a prerequisite for an efficiently running DOTS program.

The looming question over India is not just the lack of funds but the source of funds. Will the change in source from global funds to others impact the quality of drugs (monitored by the global funds) and worsen the preexisting MDR situation in the country?

## 5. Surveillance System and Laboratory Capacity Building

The laboratory has always played a critical role in diagnosing TB and monitoring its treatment. In the new millennium, the strength of the laboratory network is often a direct reflection of the success of TB control programs. However, high burden countries struggle to provide good-quality microscopy, with access to culture and drug susceptibility testing (DST) being scarce to nonexistent. Under such scenarios laboratory strengthening is a high priority on the TB agenda, but till then it would be ideal to improve access to and utilization of existing diagnostics [15]. The difference between India and China in terms of their laboratory capacities is shown in Table 1. With increasing MDR trends China has maintained its pace by creating more culture and drug susceptibility testing (DST) laboratories while India is still holding on largely to traditional sputum smear microscopy. While sputum smear microscopy offers the advantage of relatively low turnaround time, it lacks sensitivity and is incapable of identifying drug resistance. It is extremely important that with changing times and demands we move forward to achieve integration of technological innovations in the public domain for faster, sensitive, and more accurate diagnosis [16].

China's case detection after implementation of DOTS in 1992 was around 30%. This was despite the fact that the treatment success rate was 85%. It was not hard to realize that most of the patients were lost to the private health sector of China. The MOH then made it mandatory to report to the CDC every diagnosed case of TB. Hospitals are required to refer all patients suspected of having TB or diagnosed with it to the local CDC for further evaluation and treatment. Every working day CDC staff members seek patients who fail to report to CDC within 3 days after being reported by accessing the central database. This new disease reporting system has enabled China to achieve 75% case detection and 85% cure rates in 2005 [17]. Due to the integration of private hospitals and centralization of TB treatment, China has been able to conduct a survey of TB prevalence and drug resistance at level of every district.

The earliest reports of China's national survey on TB date back to 1979 [18]. The surveillance took into account all 31 provinces of China. Whilst sputum smears and cultures were examined at county levels, the DST was performed at the national level. For the treatment and management of MDR and XDR TB patients, China is building steps in the areas of technical support, research, drug resistance surveillance, diagnosis, and cooperation. Important issues such as adverse drug reactions, enhanced laboratory network, mathematical models to analyze the cost effectiveness of management of drug resistant TB over the next decade, implementation of rapid diagnosis method, and cooperation from the private sector have been taken into consideration [19]. The DOTS plus was launched in 1995 and has been running full scale treating at least 5000 patients per year in Hong Kong alone [20]. Pilot projects have been rapidly initiated to shift to faster drug susceptibility assays like Hains MTBDR and MGIT liquid culture DST for second-line drug testing [16].

India on the other hand is yet to achieve its first national survey. Only 2 states, Gujarat and Maharashtra have implemented statewide community-based surveys [21], on the basis of which the RNTCP reports that the prevalence of MDR TB is not increasing in the country. It is only after the TDR scare in Mumbai [11] has the local RNTCP in conjunction with the national program coordinators decided to notify MDR-TB cases for Mumbai region. We still await notification of all TB cases—the issues primarily being integration of private and public sector, maintaining patient confidentiality, and the nonexistent notification system. The proposal is to bring in notification through DST labs; however, there is heterogeneity in the testing methods (solid Versus liquid) and most labs are nonaccredited.

After the initiation of DOTS plus in 2007, the RNTCP promised extensive efforts for a nationwide coverage by 2010. However, only 10 states in India have DOTS plus running at sentinel sites [22]. The issue of losing patients to the vast private sector is a huge problem in India [23]. Yet, we do not have a central reporting system and database to track TB patients and their progress. This noncentralized, unchecked business of treating TB patients is adding to the burden of MDR and XDR cases. To cater to the need of such 131,000 MDR TB cases, a total of 28 intermediate reference laboratories (IRLs) under the 4 national reference laboratories [2] are existent in the Indian system. A World Bank report states that from 2009 to 2010 a total of merely 969 cases have been started on DOTS plus treatment [24] leaving a vast majority untreated.

A national survey including an all patient database will help India to realistically measure the load and pattern of drug-resistant disease which has arisen largely due to noncompliance and through wrong categorization of TB patients [25]. Had there been a central reporting system, the health system staff need not have been dependent on patient recordings but would have evidence towards right categorization and thus correct treatment.

## 6. Surveillance in Correctional Institutes

More than 9.8 million people are held in penal institutions throughout the world mostly as pretrial detainees or as sentenced prisoners. Nearly half of this is contributed by United States (2.92 m), Russia (0.89 m), and China (1.57 m sentenced prisoners) [26]. India, at any given time, has nearly 0.35 m inmates [27]. The numbers of prisons in many countries have not kept pace with the increasing population in these countries leading to overcrowding. Overcrowding is a major cause or contributing factor to many of the health problems in prisons, most notably communicable diseases (such as TB) and mental health issues, including the use of psychoactive substances [28].

These prisoners who often go back to their communities after being released are carriers of the disease and thus assist transmission. It therefore becomes important to actively survey these institutions for disease burden. Since 1998, a TB surveillance system (A joint proposal by the MOH and the Ministry of Justice) has been put in place in all 24 correctional institutions of Hong Kong in addition to the statutory TB notification system. All sentenced prisoners are forced to undergo a chest X-ray, sputum smear microscopy and if necessary other specialized investigations in public hospitals. If found positive the inmate is immediately started on DOTS. The treatment is monitored by chest physicians who regularly visit all the prison clusters covering major prisons in Hong Kong [29].

The surveillance data indicates a stable trend of disease with 836 active TB cases being detected in the 7-year period. The highest numbers of cases (441/836) were diagnosed within 3 months of incarceration. This surveillance system has not only allowed for early identification of the disease but also assisted in reducing disease severity and prevention of transmission within prisons and back to the community.

In contrast, India exhibits weak attempts to identify, prioritize, and manage diseases in the prison. There have been no systematic studies examining these issues. Health-related interventions in prisons have not been scrutinized or evaluated [30]. Thus, the Ministry of Health and Family Welfare (MOHFW) has never attempted a surveillance of prisons to identify the prevalence of TB in these institutions. Independent studies such as the one from Hindalga, Belgaum, Karnataka reported 2% prevalence of TB in a central jail [31], while the study from Western Maharashtra observed 51.51% deaths (34/66) due to TB between 2001 and 2008 [32].

In an independent letter by the National Human Rights Commission (NHRC) to the Inspector General (Prisons)/Chief Secretaries of States/Administrators of Union Territories [27], the joint secretary had pointed out to the authorities that one of the sample studies highlighted that nearly 79% of deaths in judicial custody were a result of infection of tuberculosis. The NHRC pointed out towards lack of not only entry level but even periodical medical checkups by the government doctors.

The lack of a surveillance system in correctional institutes, mental health institutions, and others with a further absence of statutory TB notification systems is hampering TB control efforts in India. Due to missing surveillance data, we shall probably never learn to focus our efforts where they are most needed.

## 7. Malnutrition

In the early 1990s, India and China were home to more than half the preschool children in the developing world who were malnourished, as measured by being stunted or underweight. In India the incidence of stunting among children aged 0–3 years was then notably higher than in China (47 versus 32%), and underweight was three times more prevalent (52 and 17%, resp.) [33].

A few key issues addressed by the Chinese health authorities as compared to India were as follows.

- China pursued a successful poverty alleviation strategy along with rapid economic growth.
- Effective nutrition, health, and family-planning interventions were implemented at a large scale in China.

Complementary interventions such as increasing the proportion of household consuming iodized salt increased from 51% in 1990 to 95% in 2005. India's disturbing finding is the decline in the use of iodized salt from 49.3% (as per NFHS-2, 1998-1999) to 36.7% (RCH-2, 2002-3) [34]. China increased the coverage of piped water and improved sanitation to 72% and 65% of the population, respectively. The 2000 WHO and UNICEF global water supply and sanitation data indicates that 92% and 73% of urban Indian had access to improved water supply and sanitation, respectively. However description of water provision in many city case studies suggested that a much smaller proportion of people had access to safe, sufficient provisions [35]. The share of mothers in China who had completed middle school increased from 32% to 57%, and the share of illiterate women fell from 22.5% to 7% [36, 37]. The percentage of women in India who had completed middle school fell from 18% in 1983 to 10% in 2004. The adult literacy rate in India is 61% compared to China's 91% [38].

The figures for malnutrition in children in India are at a shocking 43%. Even in sub-Saharan Africa, which most people assume to have the direst poverty statistics, the average child-malnutrition rate is 28%. In an all-front effort, China cut child malnutrition by two-thirds between 1990 and 2002. Today only 7 percent of Chinese children under age 5 are underweight [39]. This means that China reached its Millennium Development Goal (MDG) by 2002, more than a decade ahead of the target year 2015 [33].

Malnutrition is an important risk factor for the development of TB. Malnutrition profoundly affects cell-mediated immunity (CMI), and CMI is the principle host defense against TB. Changes in the movement and proliferation of T-lymphocyte subpopulations in response to specific antigens, and changes in the production of key cytokines, in the formation of organized granulomas, and in macrophage activation, have been identified as important components of the process [40].

While simple effective measures like hygienic living conditions and adequate nutrition may not bring the end of TB but will definitely ease the disease burden. A literate society can take preventive measures because they tend to be more aware and hence more cautious. The government should invest more in food, water, and social security which has historically proven to reduce TB burden in different societies [41].

## 8. Rural Poor

The association of TB with poverty is well established at the population and neighborhood level, usually in relation to socioeconomic disadvantage and associated ethnicity or class [42]. Both countries are fighting the poverty problem on two fronts: the urban and rural. For both China and India, poverty rates are higher in rural than in urban areas. In addition, rural areas are still home to most of the total population, and poverty is thus concentrated in rural areas [43].

China's health system has two distinctly separate parts—rural and urban. Rural health care has three levels of provision—county, township, and village. Under the post-1979 economic reforms, rural health financing has been decentralized [42]. Though the government funds have decreased towards TB treatment in rural areas, a lot of funding from external sources has allowed China to deal with the problem of rural TB.

Two projects, the Infectious and Endemic Disease Control (IEDC) project supported by the World Bank and the MOH, were initiated in 1992 and 1993 covering 573 million people in 1208 counties. This included diagnosis and free treatment if tested positive. Projects funded by Japanese government and managed by Japan international cooperation agency and a project in Tibet and Inner Mongolia being funded by Damien Foundation, Belgium is functional [44]. Despite concerted efforts there is a vast discrepancy in the accessibility to health services in rural areas in China. This fact has been openly accepted by the MOH in China which is taking measures to improve upon the shortage and allocation of health resources in poor areas of China. Economic constraints are the major reason why rural poor in China does not access health care facilities for TB care from the government [45].

The Indian government follows the westernized hospital-based medical education and training system. The health care systems are run by the constituent states and territories in India. The federal government contributes 15% towards the total expenditure mostly through national health reforms. It is only in 2005 that the government initiated the National Rural Health Mission (NRHM) in 18 Indian states to improve basic health facilities for the rural poor. According to the 2001 census, 68.8% of Indian population lived in 640,867 villages and the remaining in urban conglomerates [46].

Access to health care centers, financial constraints, Illiteracy, strong traditional/ethnic beliefs, and lack of reach of medicines in rural areas are the primary reasons for spread of TB in rural areas [47]. There is no documented evidence for the reach of RNTCP in Indian villages. It is most often through partnerships with nongovernment organisations (NGOs) (partly supported by state governments) or through community health workers (CHWs) that DOTS is practiced. The inaccessibility of medicines, lack of timely culture, and DST will steadily add to the burden of MDR TB in rural areas. There have been few reports of NGO working towards the betterment of TB patients in the villages of India. For instance Southern Health Improvement Samity (SHIS—http://www.shisindia.org/) has been successfully working since 1982 for TB care and control. They were inducted by GOI into the RNTCP and allotted with 2 districts in West Bengal. They are the only NGO's in India accredited to run 7 TB units for the state of West Bengal. The project is not just a cure and control program but a social reform effort which is dealing with the root cause of TB in these areas—poverty, lack of education, and awareness.

The Catholic Healthcare Network is the largest group in the NGO sector with more than 5,500 health care establishments in India. Eighty five percent of these health facilities are in remote rural and tribal areas, providing medical care to communities which have not been able to access the public health services. RNTCP has recently signed an MOU with 125 such centers to ensure DOTS coverage [22]. However, rising reports of increasing MDR TB cases in tribal population are a cause of concern. For instance, Jan Adhikar Manch, an organization working for the “saharia” tribe in the rural areas of a district of Madhya Pradesh, is reporting high number of TB cases, to the extent that the village is known as the “village of Widows” [48]. The prevalence of TB and number of smear positive cases increased in the Car Nicobar Island of India from 1986 to 2002 despite implementation of NTP. The most likely reason for the increase seems to be the absence of a district TB programme with enough efficiency to sustain the gains made from the one-time initial phase of special anti-TB measures [49].

The state of Chhattisgarh is a low-intensity internal conflict ridden Indian state where 80% of population is living in rural parts, and 32% are tribal. Nine out of 18 districts are inhabited by tribal population. The state TB officer in his speech at the consultative workshop of the TB and poverty subworking group of the Stop TB partnership mentioned that “47% of TB cases are being missed by the state TB programme” [50].

These instances simply highlight the neglect of the government towards the rural areas and worse still the rural poor. It may be true that our health system strengthening is still in infancy and expecting such far-reaching goals is unrealistic, but partnerships with carefully selected functional NGO's for the given area or for that matter strengthening of the quality of NRHM service delivery will have far-reaching impact on reducing the disease burden.

## 9. Urban Poor

Until the beginning of the 1990s, poverty in China was regarded largely as a rural phenomenon, and the rural poor were the focus of antipoverty policies [51]. However, in the 1990s, urban poverty came to be seen as a problem that potentially threatened a substantial percentage of the urban population. Unlike in the past, the government has not been able to provide the urban labor force with a job guarantee.

To respond to the increasingly urgent problem of urban poverty, several urban social assistance and protection schemes have started to emerge since 1997 covering only the urban residents, including a support program for laid-off employees like the Minimum Living Standard Scheme (MLSS) or *di bao* scheme, Medical Financial Assistance (MFA) to poor households, and the recently established social assistance stations targeted at rural migrants. Each city decided on its own poverty line depending upon the living standard of the city. The MLSS is mainly handled by the city government as per the decided poverty line [51].

One of the major deficiencies of the *di bao* program is the lack of coverage of health care. To remedy this problem, several cities have supplemented the *di bao* scheme with various types of ad hoc medical assistance programs [52].

These programs, although they vary across localities, in general provide a partial or full waiver for inpatient and outpatient services, or subsidies to enroll in medical insurance schemes for the population eligible for* di bao* [51].

In 2004, the Chinese government along with the UK Department for International Development (DFID) cofounded a national project named as the Urban Health and Poverty Project (UHPP). The project focuses on urban health reform and poverty alleviation and is also the country's largest ongoing community health and medical aid programme [53] which covers cost of TB treatment of several patients.

India is yet to put a number to the poor surviving in urban areas of the country in the past 10 years. The survey began in June 2011 and was expected to be completed by December 2011. This is important in the context of the proposed Food Security Act and the Rajiv Awas Yojana (RAY) which aims to make cities free of slums besides better targeting of other schemes. It is not surprising that since demographic and income data are incomplete, social data is even more scarce [54].

Across the country the poverty line is decided on the basis of daily consumption and expenses per head at INR 20 (US $0.38). Policies for urban poor have been in existence since the 1950's, but the primary objective has been urban development and housing. Unlike rural areas which have an organized 3 tier health delivery structure, the urban area has no such structure available resulting in poor health indicators of urban poor.

A new health insurance scheme for the Below Poverty Line (BPL) families in the unorganized sector was formally launched on October 1, 2007 named as Rashtriya Swasthya Bima Yojna (RSBY). There is a five-year plan for rolling out the RSBY which allows each participating state to contract 20% of their respective districts each year. On one hand, the government is launching ambitious schemes for health of urban poor, and on the other hand it is minimizing the number who can access it by deciding on a value of INR20 as the poverty line cutoff (http://www.rsby.in/).

India's ambitious national programme to provide quality healthcare to the country's urban poor—the National Urban Health Mission (NUHM) which is moribund with absence of design and approach—has been shelved for the time being and will not be launched during the present 11th five-year plan. The government proposes to launch it in the 12th five-year plan (2012–2017).

## 10. Spurious Drugs

**Figure 1:**
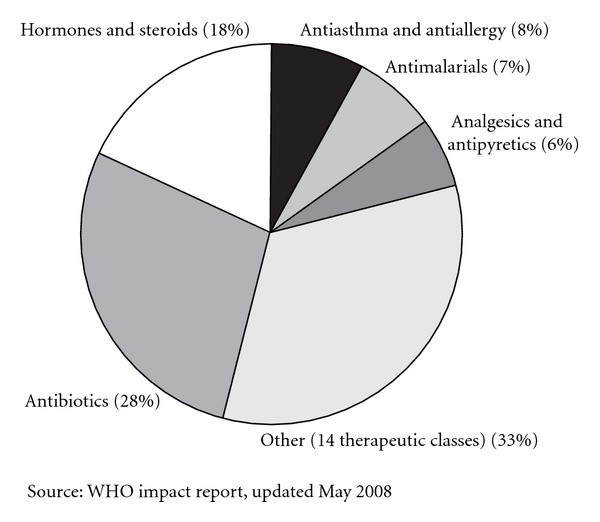
Reports of counterfeit drugs by therapeutic class received by WHO between 1999 and 2002, adapted from International policy network repost [4]. Prevalence of fake medicines is seen across all classes of drugs, a large proportion of them being antibiotics.

Counterfeit and substandard drugs are a serious and growing problem around the world—especially in less-developed countries (LDCs) (Figure 1). There are many reasons for this, including imitation, inappropriate packaging, poor manufacturing processes, and improper conditions during transportation and storage [55].

The scale of the problem remains unclear. The World Health Organisation (WHO) estimates that counterfeit drugs constitute up to 25 per cent of the total medicine supply in LDCs. In Africa and South East Asia, more detailed sampling found that between 30 and 60 per cent of medicines were substandard [4].

While 10 and 30% of all pharmaceuticals in developing countries are counterfeit, (2006 WHO figures cited in the Organization for Economic Cooperation and Development (OECD) report), India (35%) is the biggest culprit in the spurious drugs market alongwith Egypt (7%) and China (6%).

Studies have estimated that around 700,000 malaria and tuberculosis deaths per annum are attributable to fake drugs. Drug marketing surveys indicate that 94 million dollars worth of antituberculous drugs were purchased in India in the year 2006; approximately 75% of these drugs were procured from the private sector [23]. This indicates that the quality of the drugs may or may not be as per internationally recommended and accepted standards also increasing the likelihood of MDR TB.

Another study highlighting the size and characteristics of private market in High Burden Countries (HBCs) found that while the size of India's private drug market for TB treatment was large (capable of treating around 117% of all estimated incident TB cases), China has a medium-sized market (capable of treating around 23% of all estimated incident TB cases). While India's private market share is largely dominated by fixed-dose combinations, China's private drug market is predominated by loose drugs [56] (Table 2).

In separate studies, it was found that in the state of Kashmir in India the rate of MDR-TB had increased which was attributed to the fake drug—purchased over the counter over a prescription from the private practitioner. The study conducted by the states' premier research institute Sher-e-Kashmir Institute of Medical Sciences, reported lack of activity of the anti-TB drugs procured over the counter leading to high levels of MDR in the state [57].

A study conducted by randomly sampled antimalarial, antibiotic, and antimycobacterial drugs collected from pharmacies in urban and periurban areas of Delhi and Chennai, India found that 12.6% of drugs procured were spurious [58].

WHO reports that counterfeit or poor-quality anti-TB drugs are easily available in the open market. The MOH has three options: lobby for legislation that prohibits the sale of anti-TB drugs without a doctor's prescription; accredit doctors who are trained to treat MDR-TB, and apply to the Green Light Committee for access to quality-assured second line medication [59].

At the extreme end of this scale, India and China have introduced the death penalty for certain offences involving counterfeit drugs though so far only China has actually invoked the penalty [55].

Though both countries have offered promises to check on their spurious drug stores, both are finding it difficult to take responsibility and thus necessary action. But if India and China wish to continue with pharmaceutical growth adding to their economic growth, then the 2 countries need a disease-free generation and strict measures to curb counterfeiting of drugs.

## 11. Private Practice and TB

While China's health services are primarily finan

ced by out-of-pocket spending (private financing), health care providers, especially the hospital industry, are still dominated by state ownership and government control (public provision) [60]. Data collected initially revealed that private sector provision of health services in China is still small and lacks sophistication [61]. Although private practitioners are gaining roots, they are relatively rare (accounting for only 3% hospitals) in the 3 Chinese provinces of Guangdong, Shanxi, and Sichuan [62].

 In a study conducted in Hong Kong, it was found that of a total of 6262 notified tuberculosis patients in 2004, 1662 (26.5%) were recruited into the study; of these, 42.6% first presented to private doctors, and 57.4% to the public sector. It was observed that doctors in the public sector tend to take a Chest X-ray (CXR) and a sputum examination more often than their private counterparts. The referral time delay from private sector varied widely with 11% referred without delay, 60% by 1 month, and 17% after 3 months. These marked differences very likely reflect different clinical practices or availability of laboratory support among the various health care sectors [63].

In another study aimed to obtain details of management by private practitioners, and in particular of the antituberculosis chemotherapy, prescribed fewer patients had a sputum examination done but had a CXR when they attended to a private clinic. Both the sputum smear-positive (65%) and -negative cases (71%) were told that they might have TB. When patients recalled their prescriptions or samples, it was found that only 19% of the cases were definitely or probably prescribed an antituberculosis regimen although this was not always an adequate regimen [64].

The problem with the private sector and TB is not so much an issue with China as it is with India, for 2 primary reasons, private health sector has less scope in China, and, secondly, the centralized system of TB notification brings all TB cases under the national policy.

India has a huge, unwieldy, and poorly regulated private medical sector with an estimated 10 million registered doctors, with a ratio of 1 doctor per 1000 population, far in excess of the WHO guideline of 1 in 3000. Around 50% of these are qualified and registered non-allopathic doctors practicing alternative systems of medicine such as homeopathy, Ayurveda, and Unani 

[23]. While it has been reported that a majority of patients do not access any health care systems [65], nearly 50–70% of tuberculosis patients in India continue to prefer private healthcare. These patients are not monitored by the RNTCP and, therefore, do not benefit from the potential success rates offered by DOTS [23].

The increasing trend of MDR in samples, sent to the laboratory at a reputed tertiary care private hospita

l, gave rise to study to evaluate the prescription practices of the private practitioners in Mumbai, in particular the slums of Dharavi. It was found that the participating practitioners had never been approached or oriented by the local TB programme. Two independent studies highlight this situation. In 1991 a study found that 102 private doctors practicing in a slum in the former “Bombay” showed a lack of awareness towards regimens for TB therapy; that is, 100 private doctors prescribed 80 different regimens [66]. Nearly, two decades later another study recorded only 6 of the 106 respondents writing a prescription with a correct drug regimen. Here 106 doctors prescribed 63 different drug regimens [67]. There was a tendency to overtreat with more drugs for longer durations. Only 3 of the 106 respondents could write an appropriate prescription for treatment of multidrug-resistant TB. These may be single isolated reports but the rising number of slums in the country and an equivalent rise in the number of private medical practitioners with no regulation from the government is a warning in itself [68].

Originally, DOTS was primarily implemented through NTPs. It was recognized, however, that health systems are pluralistic and that private practitioners (often general practitioners) functioning in isolation from NTPs were an important source of care for many patients though their services did not meet international standards [69]. This fact gave rise to the strategy of public-private mix (PPM) DOTS.

PPM DOTS is now known as “PPM for TB Care and Control” and is a core component of the WHO STOP TB Strategy, entitled “Engage All Care Providers” [70]. Fifty-eight of 93 countries had PPM activities in 2008. In China, India, Nigeria, and the Philippines, PPM contributed to detecting more than 25% TB cases while maintaining high treatment success rates [71]. Independently, China has contributed through PPM (mediated via general public hospitals) to identification of 15% of all notified tuberculosis cases countrywide whereas India has contributed to 36% of new sputum smear-positive cases in 14 selected cities bearing a population of 50 million [71]. These figures are not in India's favour because a previous report evaluating the effect of PPM launch in India reported 57% notification of TB cases from 14 initiative centers with a population of 20 million at the end of August 2004 [72]. This is also indicative of the fact that despite schemes and funds in place, we cannot ensure 

the functioning of the scheme because there is no followup and insistence to pursue it stringently.

Both India and China need to reassess their stand on PPM and take it forward. With changing mind sets and population inclined towards the private sector in both countries, it may be to the advantage of DOTS and the government to implement PPM more comprehensively as a combination approach of incentives and regulation.

## 12. Conclusion

The economic burden of TB in developing countries is not just on treatment of patients but also a national income loss by a recurrently sick population. While India continues to live in denial of the problem, China has forged ahead by not only decreasing TB incidence, but also generating a task force competent in handling future health emergencies. As per WHO, China has one of the most successful TB control programs. The fact that has added to the success of the NTP in China is the political will and commitment that the Chinese government has displayed. Integration of services, a central database for case reporting and treatment, enhanced surveillance, use of high end techniques like Hains MTBDR plus for identification of drug resistance, increased laboratory, and surveillance network, procurement of funds from every possible source and its optimum utilization has added to the credibility of the government.

India on the other hand has to begin with the acceptance of the menace that TB poses. The annual health reports (feel good mirages) which are still mandates and commitments should increasingly depict results which are free from convoluted targets. The administration and public health system must first acknowledge that facts and figures are not a burden but signs of a disease burden. As India continues to pledge its commitment, it is only wasting further time before the disease reaches epidemic proportions. India first needs a nation-wide surveillance to correctly identify the number of TB-affected patients. They have to be distributed in every category so as to identify the targets and move progressively towards achieving them. HIV/MDR/XDR will just add to the existing burden and make treatment more expensive, ill managed, and cumbersome.

It is also time, that we bring all TB cases under a central cover, which not only identifies the burden from time to time but also makes it mandatory for private practitioners to have the same treatment approach as the DOTS. The poor, undernourished, and immune suppressed population needs special attention. Like China, India needs a definite and massive boost to its public health system through rapid increase of human resource, infrastructure, and population outreach. We would conclude that TB is so omnipresent that only a decedent civilization/government would want to overlook it.

## Figures and Tables

**Table 1:** Microbiology facilities 2010 (adapted from WHO report 2009) [2].

Laboratories	India	China
Smear (/100000 population)	1.1	0.2
Culture (/5 million population)	0.1	3.5
DST (/10 million population)	0.2	1.2

**Table 2:** Size and characteristics of private TB market, adapted from Wells 2011.

Country	Incident cases (2008)	Coverage by first line private sector drugs*	% change in volume 2004–9	% of private market that is loose drugs	Number of manufacturers with 0.3% of private first line market share	Fluoroquinolone coverage of incident MDR-TB cases^#^	Fluoroquinolone coverage of all incident cases^&^
India	1,982,628	117%	−3	23%	6	41%	6.1%
China	1,301,322	23%	59	98%	9		

*% of all incident cases that can be treated by first line drugs in private market (average across 4 first line drugs, assuming daily 6–8 month regimen). Data for this and other columns, unless noted, are for Q4 2008–Q3 2009.

^#^Assuming daily dosing for 18 month regimen, and no use for drug-sensitive TB.

^&^Assuming daily dosing for 6 month regimen, and no diagnosis of drug-resistant TB.
